# Sulfur-Doped ZnO as Cathode Interlayer for Efficient Inverted Organic Solar Cells

**DOI:** 10.3390/ma18081767

**Published:** 2025-04-12

**Authors:** Ermioni Polydorou, Georgios Manginas, Georgios Chatzigiannakis, Zoi Georgiopoulou, Apostolis Verykios, Elias Sakellis, Maria Eleni Rizou, Vassilis Psycharis, Leonidas Palilis, Dimitris Davazoglou, Anastasia Soultati, Maria Vasilopoulou

**Affiliations:** 1 Institute of Nanoscience and Nanotechnology (INN), National Center for Scientific Research (NCSR) Demokritos, 15341 Agia Paraskevi, Greece; e.polydorou@inn.demokritos.gr (E.P.); zoegeorgiopoulou@gmail.com (Z.G.); e.sakellis@inn.demokritos.gr (E.S.); m.rizou@inn.demokritos.gr (M.E.R.);; 2 Department of Mechanical Engineering, School of Engineering, University of West Attica, 12244 Egaleo, Greece; 3 Solid State Physics Section, Department of Physics, National and Kapodistrian University of Athens, Panepistimioupolis, 15784 Zografos, Greece; 4 Department of Physics, University of Patras, 26504 Patras, Greece

**Keywords:** zinc oxide, sulfur doping, electron extraction layer, inverted organic solar cells

## Abstract

Bulk heterojunction (BHJ) organic solar cells (OSCs) represent a promising technology due to their cost-effectiveness, lightweight design and potential for flexible manufacturing. However, achieving a high power conversion efficiency (PCE) and long-term stability necessitates optimizing the interfacial layers. Zinc oxide (ZnO), commonly used as an electron extraction layer (EEL) in inverted OSCs, suffers from surface defects that hinder device performance. Furthermore, the active control of its optoelectronic properties is highly desirable as the interfacial electron transport and extraction, exciton dissociation and non-radiative recombination are crucial for optimum solar cell operation. In this regard, this study investigates the sulfur doping of ZnO as a facile method to effectively increase ZnO conductivity, improve the interfacial electron transfer and, overall, enhance solar cell performance. ZnO films were sulfur-treated under various annealing temperatures, with the optimal condition found at 250 °C. Devices incorporating sulfur-doped ZnO (S-ZnO) exhibited a significant PCE improvement from 2.11% for the device with the pristine ZnO to 3.14% for the OSC based on the S-ZnO annealed at 250 °C, attributed to an enhanced short-circuit current density (J_sc_) and fill factor (FF). Optical and structural analyses revealed that the sulfur treatment led to a small enhancement of the ZnO film crystallite size and an increased n-type transport capability. Additionally, the sulfurization of ZnO enhanced its electron extraction efficiency, exciton dissociation at the ZnO/photoactive layer interface and exciton/charge generation rate without altering the film morphology. These findings highlight the potential of sulfur doping as an easily implemented, straightforward approach to improving the performance of inverted OSCs.

## 1. Introduction

Over the past two decades, bulk heterojunction (BHJ) organic solar cells (OSCs) have emerged as a breakthrough technology for optoelectronic applications due to their unique advantages of their low-cost production, solution processability, lightweight and flexible manufacturing capabilities which can be further extended to roll-to-roll processing [1,2,3,4,5]. Currently, the power conversion efficiency (PCE) of optimized single-junction OSCs with reduced photon and carrier losses has reached the benchmark of 20% and, very recently, an impressive certified PCE of 20.82% was demonstrated [6]. In addition to the development of novel photovoltaic materials with higher absorption efficiency and charge mobilities [7,8,9,10,11,12,13,14], ingenious interfacial engineering is identified to be of paramount importance, due to its fundamental role in addressing the inherent limitations of the energy-level alignment at the various interfaces and enhancing the charge extraction [15,16,17]. OSCs were initially developed using conventional structures, where a bulk heterojunction layer, composed of a blend of electron-donor (p-type) and electron-acceptor materials, was positioned between a transparent conductive metal oxide anode, usually indium tin oxide (ITO), and an opaque low-work-function metal cathode [18,19,20,21]. In the pursuit of high-performance OSCs, it was realized that the selection of proper interfacial layers was crucial for the energy-level alignment at metal/active layer interfaces in order to ensure an efficient charge transfer/extraction. On the anode electrode side, poly(3,4-ethylenedioxythiophene)–poly(styrene sulfonate) (PEDOT:PSS) was commonly employed as a hole injection/transport layer to modify ITO and facilitate hole injection. However, the acidic and hydroscopic nature of PEDOT could cause the corrosion of the ITO electrode [22,23,24], promoting moisture penetration into the photoactive layer, thus leading to the rapid degradation of the solar cell. In view of the above adverse issues, an inverted architecture was introduced featuring a low-work-function electron extraction layer (EEL) modified cathode and a stable high-work-function anode. Compared with the conventional OSC structure, the inverted one exhibited higher environmental stability and photostability with slower degradation rates [25,26].

In inverted OSCs, the primary role of the EEL is to prevent charge recombination between the photoactive layer and the bottom electrode, as well to facilitate electron extraction towards the cathode electrode. Accordingly, it is imperative that this layer exhibits a wide optical bandgap, high optical transparency in the visible region and sufficient electrical conductivity [7,8,9,10,11,12,13,14,15,16,17,18,19,20,21,22,23,24,25,26,27,28,29]. Among various interfacial materials, zinc oxide (ZnO) is one of the most commonly employed EELs in the inverted OSCs, exhibiting several inherent merits, including tunable optoelectronic properties, excellent optical transparency, low toxicity and high electron mobility [29,30]. Moreover, simple solution-processing methods can be applied to develop ZnO-based EELs at relatively mild conditions; thus, they are well suited for large-scale manufacturing on flexible substrates [31,32]. However, low-temperature solution-processed ZnO often suffers from the high density of surface defects, such as oxygen vacancies (V_O_s) (or zinc dangling bonds) and zinc vacancies (V_Zn_s) (or oxygen dangling bonds) [33,34,35]. Such surface defects act as effective recombination centers for photogenerated charge carriers, hindering the charge collection efficiency, thus decreasing the photocurrent and power conversion efficiency as well as the device lifetime. Consequently, the effectiveness of ZnO layers in inverted OSCs strongly depends on the bulk and/or surface traps and defects. Hence, there has been a general consensus to enhance the electron conductivity/mobility and transport in ZnO by passivating these undesirable defects. In this context, numerous studies have been aimed at modifying the surface of ZnO films, boosting the PCE and lifetime of inverted OSCs.

An effective approach for the passivation of the ZnO surface defects, in particular, is to introduce appropriate interfacial modifiers, such as self-assembled monolayers (SAMs) [36,37,38,39], small molecules [40,41], conjugated polyelectrolytes (CPEs) [42,43,44], alcohol- or water-soluble conjugated polymers [45,46,47] and ionic liquids (ILs) [48,49], between ZnO and the photoactive blend. Additionally, the gas plasma treatment [50,51] of ZnO or the insertion of ultrathin ALD metal oxides [52,53] prior to the deposition of the photoactive blend effectively addresses and tackles the defect-related problems, suppressing the unwanted non-radiative interfacial charge recombination. Another employed strategy is the exposure of the ZnO film to UV light, a process known as the “light-soaking” method, where UV-generated charges fill the traps in the ZnO film (due to the presence of chemically adsorbed oxygen on its surface, trapping negative charges) increasing its n-type conductivity [54,55]. Doping is also considered an efficient way to passivate surface defects, hampering the adsorption of oxygen while improving the n-type conductivity of ZnO [56,57,58]. The development of doped ZnO cathode interfacial layers via group-II [59,60] and group-III [61,62,63,64] elements has been proven to be of utmost significance in fabricating highly efficient inverted OSCs with enhanced stability. Several studies have reported the incorporation of sulfur (S) atoms into the ZnO lattice through substitutional doping, replacing oxygen atoms. These efforts were mainly focused on enhancing ZnO’s photocatalytic activity under visible light and improving its electron transport properties [65,66]. In a work by Zafar et al. [67], the effect of sulfur doping in the ZnO layer via sol–gel chemistry was investigated on the performance of inverted OSCs. It was found that doping with sulfur, by adding thiophene to the ZnO solution as a sulfur dopant, increased the short-circuit current density (J_sc_) and fill factor (FF) values of the fabricated solar cells. This improvement was attributed to the slight bandgap narrowing at the interface between the ZnO EEL and the photoactive blend, caused by S atoms filling the oxygen vacancy sites within the ZnO lattice structure.

In our work, an alternative approach to modify the ZnO surface and, thereby, its electrical properties through sulfur doping is proposed by employing a simple post-annealing step in a sulfur-rich environment. An in-depth study of the mechanism responsible for the enhanced inverted OSCs performance is also demonstrated. Therefore, inverted OSCs based on the photoactive layer (poly[4,8-bis(5-(2-ethylhexyl)thiophen-2-yl)benzo[1,2-b;4,5-b′]dithiophene-2,6-diyl-alt-(4-(2-ethylhexyl)-3-fluorothieno[3,4-b]thiophene-)-2-carboxylate-2-6-diyl]:3,9-bis(2-methylene-((3-(1,1-dicyanomethylene)-6,7-difluoro)-indanone))-5,5,11,11-tetrakis(4-hexylphenyl)-dithieno[2,3-d:2′,3′-d′]-s-indaceno[1,2-b:5,6-b′]dithiophene) (PCE10:IT-4F) incorporating ZnO or sulfurized ZnO (S-ZnO) treated under different annealing temperatures as EELs were fabricated. The inverted OSC with the S-ZnO film annealed at a moderate temperature (250 °C) during the sulfurization process exhibited a higher power conversion efficiency of 3.14%, representing a 49% improvement compared to the reference device (2.11%) using pristine ZnO as the EEL. On the other hand, as the annealing temperature is increased, the amount of oxygen vacancies in the S-ZnO films annealed at 350 °C and 450 °C increased, leading to excessive amounts of defects that may act as charge carrier traps degrading the performance of OSCs using S-ZnO films as EELs. The increase in the overall device performance of the S-doped inverted OSCs was investigated using several material and device characterization methods, including UV-Vis optical absorption spectroscopy, infrared spectroscopy, steady-state and transient photoluminescence (PL) measurements, X-ray diffraction (XRD, energy dispersive X-ray (EDX) spectroscopy, atomic force microscopy (AFM), electrical conductivity and contact angle measurements. It was found that the incorporation of sulfur (S) into the ZnO lattice in a substitutional mode altered the structural and surface properties of the ZnO films. Under optimized conditions (annealing temperature at 250 °C for 30 min during sulfurization process), the S-doped ZnO sample exhibited improved conductivity and enhanced interfacial exciton dissociation and charge collection efficiency. Both the decreased charge carrier recombination as well as the smooth surface of the photoactive film spin-coated on top of the S-ZnO (250 °C) layer resulted in the enhancement of the inverted OSCs’ performance.

## 2. Materials and Methods

### 2.1. Preparation of ZnO Films

Solution-processed 50 nm thin ZnO films were prepared following a sol–gel method and a two-step annealing procedure using zinc acetate dihydrate in 2-methoxyethanol–ethanolamine as a precursor solution. The solution was stirred for 2 h at 60 °C using a magnetic stirrer to obtain a homogeneous solution. Next, it was spin-coated at 2000 rpm and immediately annealed at 85 °C for 10 min. Then, the substrates were further annealed at 250 °C for 30 min. Zinc acetate dihydrate was purchased from Sigma-Aldrich (St. Louis, MO, USA) (≥98%, CAS number: 5970-45-6) and used without further purification.

### 2.2. Sulfurization Process

Sulfurization of S-ZnO films at various temperatures (250 °C, 350 °C and 450 °C) was performed in a one-zone furnace system equipped with a 2″ quartz tube. The samples were placed in the middle of the tube, while 100 mg of sulfur powder (purchased from Sigma-Alrich) loaded in a ceramic boat was positioned at a lower temperature zone (200 ± 2 °C) 14 cm away from the sample. The tube was first heated to 100 °C with Ar/H_2_ (a mixture of 95% Ar and 5% H_2_) with a flow rate of 900 sccm in order to remove oxygen and moisture from the furnace. Then, the temperature was further increased to the desired temperature with a heating rate of 10 °C/min. Each S-ZnO thin film was obtained by sulfurization of the corresponding ZnO sample at the desired temperatures for 30 min and an Ar/H_2_ flow rate of 100 sccm. Finally, the sulfurization process was completed with natural cooling down to room temperature.

### 2.3. Device Fabrication

Inverted OSCs were fabricated on indium tin oxide (ITO)-coated glasses which were purchased from Sigma-Aldrich (CAS number: 50926-11-9) and served as cathode electrodes. Substrates were ultrasonically cleaned with a standard solvent regiment of 15 min each in deionized water, acetone and 2-propanol and dried with N_2_ gas after each bath, respectively. The ZnO and/or sulfurized ZnO layer were then deposited followed by deposition of PCE10:IT-4F (poly[4,8-bis(5-(2-ethylhexyl)thiophen-2-yl)benzo[1,2-b;4,5-b′]dithiophene-2,6-diyl-alt-(4-(2-ethylhexyl)-3-fluorothieno[3,4-b]thiophene-)-2-carboxylate-2-6-diyl]:3,9-bis(2-methylene-((3-(1,1-dicyanomethylene)-6,7-difluoro)-indanone))-5,5,11,11-tetrakis(4-hexylphenyl)-dithieno[2,3-d:2′,3′-d′]-s-indaceno[1,2-b:5,6-b’]dithiophene) as the photoactive layer from a solution with a concentration of 20 mg/mL (1:1.25 in chlorobenzene, CB (Sigma-Aldrich, 99.9%, CAS number: 108-90-7), with 0.5% DIO (Sigma-Aldrich, CAS number: 24772-63-2), spin-coated at 1200 rpm for 90 s and then annealed at 150 °C for 10 min. Note that all depositions and thermal treatments of the photoactive layers were carried out in environmental conditions. Then, an approximately 30 nm thick sub-stoichiometric molybdenum oxide (MoO_x_) layer was deposited on top of the photoactive layer to serve as the hole extraction layer. The devices were completed with a 150 nm thick aluminum film serving as the anode electrode and deposited in a dedicated thermal evaporator at a pressure of 10^−6^ Torr through a shadow mask, which defined the device active area to be equal to 12.56 mm^2^. The devices were then measured in air at room temperature without additional encapsulation. IT-4F and PCE10 were purchased from Ossila (Sheffield, UK) (CAS number: 2097998-59-7 and 1469791-66-9, respectively) and the rest of the chemicals were purchased from Sigma-Aldrich (acetone ≥ 99.5% CAS number: 67-64-1 and 2-propanol ≥ 99.5% CAS number: 67-63-0) and used with no further purification.

### 2.4. Characterization Techniques

Absorption and transmittance spectra were measured using a PerkinElmer Lambda 40 UV-Vis spectrophotometer (Spectralab Scientific Inc., Markham, ON, Canada). Fourier transform infrared (FTIR) transmission spectra of ZnO films were obtained on a Bruker Tensor 27 spectrometer (Bruker, Billerica, MA, USA) (at 4 cm^−1^ resolution, 64 scans) with a DTGS detector (Sciencetech, London, ON, Canada). The thickness of all films was measured using FR-pRo UV/NIR-HR (SPS Polos, Putten, The Netherlands) operating in 190–1100 nm spectral range, capable of measuring film thickness in the 1 nm–100 μm spectral range. Elemental composition identification was carried out using scanning electron microscopy (SEM) (Thermo Fisher Scientific Inc., Waltham, MA, USA). Specifically, a variable pressure FEI Quanta microscope (FEI Company, Hillsboro, OR, USA) equipped with an EDAX Energy Dispersive X-ray Spectroscopy (EDS) detector (AMETEK, Inc., Berwyn, PA, USA) was employed. The X-ray diffraction analysis of the films was conducted using a Rigaku Smart Lab Diffractometer (Rigaku SmartLab, Neu-Isenburg, Germany) with Cu-Ka radiation. Θ/2Θ scans were employed and the angular range for data collection was 2.0–80.0°, scanned in steps of 0.03° with a scan speed of 0.3 s/step. The surface morphology of the films was recorded with an NT-MDT AFM system (LaborScience SA, Athens, Greece) in tapping operation mode. Photoluminescence measurements were conducted with a green diode laser (532 nm wavelength) for sample illumination at a 45° angle through a focusing lens. The diameter of the illuminated area was approximately 2 mm, and the laser power on the sample was approximately 2 mW. Current density–voltage characteristics of the fabricated solar cells were measured with a Keithley 2400 source-measure unit. Cells were illuminated with an Xe lamp and an AM 1.5G filter to simulate solar light illumination conditions with an intensity of 100 mW/cm^2^ (1 sun), as recorded with a calibrated silicon photodiode.

## 3. Results and Discussion

**Figure 1:**
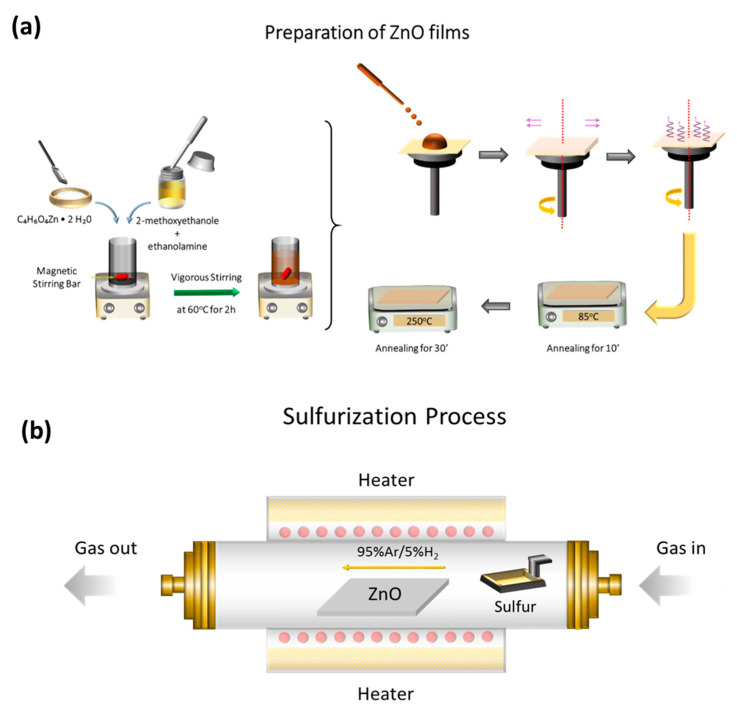
(**a**) Schematic diagram of deposition process of ZnO. (**b**) Schematic diagram of sulfurization furnace.

Solution-processed zinc oxide (ZnO) thin films were deposited on indium-tin oxide (ITO) substrates through spin-coating. Then, ZnO films were subjected to sulfurization in various annealing temperatures, as described in detail in the Experimental Section, forming sulfur-doped ZnO films, named hereafter as S-ZnO. Figure 1a,b present the schematic illustration of the solution-based preparation of ZnO films and the sulfurization process, respectively. Note that the sulfurization process did not affect the thickness of the ZnO and S-ZnO, as measured using FR-pRo UV/NIR-HR operating in the 190–1100 nm spectral range, capable of measuring the film thickness in the 1 nm–100 μm spectral range.

**Figure 2:**
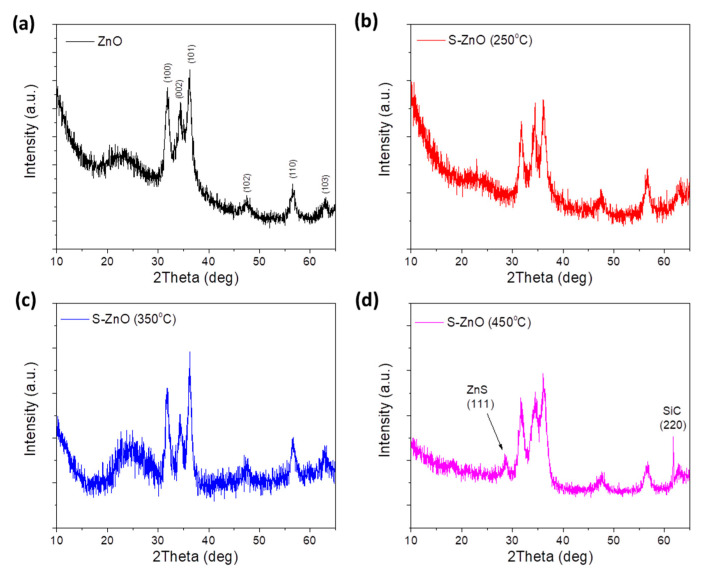
XRD diffractograms of (**a**) pristine and S-doped ZnO samples annealed at (**b**) 250 °C, (**c**) 350 °C and (**d**) 450 °C for 30 min.

Figure 2a–d shows the XRD diffractograms of the pristine and S-doped ZnO films annealed at 250 °C, 350 °C and 450 °C for 30 min during the sulfurization process, respectively. In all cases, the characteristic peaks of the hexagonal ZnO wurtzite phase corresponding to the (100), (002), (101), (102), (110) and (103) planes are observed. The absence of characteristic ZnS peaks in the case of the S-ZnO (250 °C) and S-ZnO (350 °C) implies that sulfur atoms are primarily incorporated on the surface of ZnO films without significantly affecting the ZnO lattice. On the other hand, a peak at 28.49° appears in the S-ZnO annealed at 450 °C, which is attributed to the ZnS (111) crystallographic plane [68]. Furthermore, a small increase in the d-spacing of the (100) and (101) planes is observed upon S doping, suggesting longer inter-planar distances in the S-ZnO samples. More importantly, thermal annealing during the sulfurization process reduces the intensity of the XRD peak, although it enhances the crystalline size of the ZnO film, as indicated by the reduced FWHM of the (100), (002) and (101) peaks in the S-doped ZnO samples (Table S1). These changes could be attributed to the incorporation of S in the lattice of the ZnO, due to the larger ionic radius of S^2−^ than that of O^2−^ [69]. The d-spacing and the crystalline size (*D*) were estimated by Bragg Equation (Equation (1)),

(1) nλ=2dsinθ

and Scherrer formula (Equation (2)),

(2) $$D=\frac{0.9\lambda }{FWHM\cos\theta }$$

respectively, where *λ* is 0.154 nm and is summarized in Table S1.

A more detailed evaluation of the structural properties of the pristine and S-doped ZnO samples was also carried out, calculating the lattice constants α and c, as well as the volume (*V*) of the unit cell and the length (*L*) of the Zn–O bond using Equations (3)–(7) [70] (Table S2).

(3) $$\alpha_{100}=\frac{\lambda }{\sqrt{3}\sin\theta }$$

(4) $$c_{002}=\frac{\lambda }{\sin\theta }$$

(5) $$V=\frac{\sqrt{3}}{2}a^{2}c$$

(6) $$u=\frac{a^{2}}{3c^{2}}+0.25$$

(7) $$L=\sqrt{\left(\frac{a^{2}}{3}\right)+{\left(\frac{1}{2}-u\right)}^{2}c^{2}}$$

As the annealing temperature increases the volume of the unit cell increases, indicating a better incorporation of S atoms in the ZnO lattice. S-doping also results in higher lattice parameters, demonstrating a lattice expansion attributed to the incorporation of an adequate amount of S atoms in the lattice of the ZnO in a substitutional mode. Furthermore, the S-doped samples exhibit higher Zn–O bond lengths compared with the pristine ZnO, which is assigned to the higher ionic radius of S^2−^ ions.

**Figure 3:**
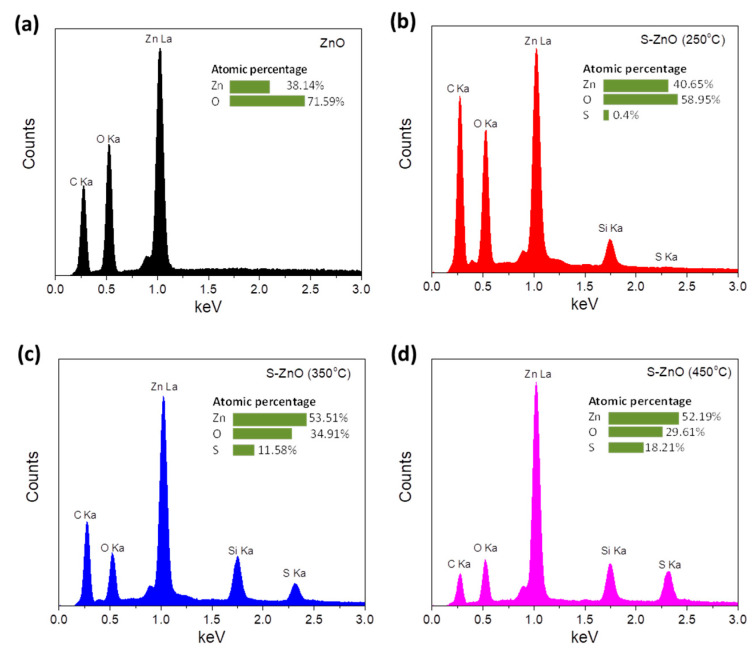
EDS of (**a**) pristine and S-doped ZnO samples annealed at (**b**) 250 °C, (**c**) 350 °C and (**d**) 450 °C for 30 min.

In order to further investigate the presence of sulfur in the ZnO samples subjected to sulfurization upon annealing at various temperatures, energy dispersive X-ray spectra (EDS) were recorded. Figure 3a–d shows the typical EDS of the pristine ZnO S-ZnO annealed at 250 °C, 350 °C and 450 °C for 30 min, respectively, while the atomic percentages analysis is shown as inset. Table S3 also summarizes the energy dispersive X-ray spectroscopy (EDX) analysis of the same samples. All S-doped samples exhibit an oxygen (O) peak at 0.53 keV, a zinc (Zn) peak at 1.02 keV and a sulfur (S) signal at 2.31 keV, proving the efficient doping of the ZnO with S. An increase in S-doping upon increasing the annealing temperature during sulfurization is also observed (the insets of Figure 3 show the atomic percentage of the elements Zn, O and S). The EDX and XRD analysis show that the sulfurization of ZnO results in the substitution of sulfur at the oxygen sites.

The substitutional mode in the S-ZnO samples will also affect the FTIR bands. Figure S1a shows the FTIR spectra of thick ZnO and S-ZnO films. The characteristic Zn–O band appears at 450 cm^−1^ in both cases, while a very small peak at 414 cm^−1^, attributed to symmetric Zn-S stretching, appears in the spectrum of the S-ZnO film (Figure S1b). In addition, the Zn–O band is slightly shifted towards lower wavenumbers in the S-ZnO (250 °C) sample, which could be related to the higher length bond compared to the ZnO sample, as revealed from the XRD measurements. This is attributed to the substitutional S-doping of the ZnO [71]. Furthermore, the band attributed to the O-H bending centered at ~3400 cm^−1^ is significantly reduced in the S-ZnO case due to the annealing of the ZnO film during the sulfurization process.

**Figure 4:**
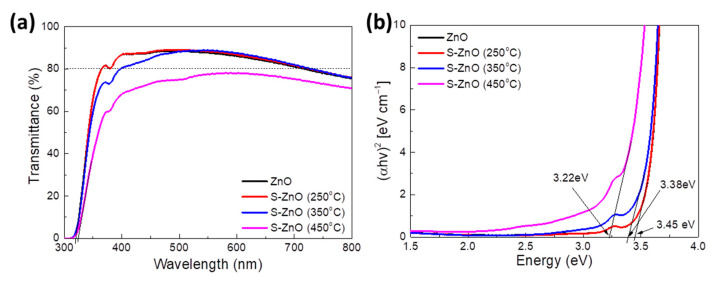
(**a**) UV-Vis transmittance spectra and (**b**) Tauc plots of ZnO and S-ZnO annealed at various temperatures upon sulfurization.

To examine the optical properties of ZnO with sulfur doping, UV-Vis spectroscopic measurements were performed. The UV-Vis transmittance spectra of the pristine ZnO and sulfur-doped ZnO (S-ZnO) films annealed at different temperatures, 250 °C, 350 °C and 450 °C, are shown in Figure 4a. It is observed that the pristine and S-doped ZnO films annealed up to 350 °C are highly transparent in the visible region, maintaining their transmittance over 80%. On the other hand, when the annealing temperature increased to 450 °C during the sulfurization process, the S-ZnO film exhibited a lower transmittance, likely due to the enhanced thermally activated electronic conductivity of the S-doped ZnO.

Figure S2 of the Supporting Information presents the absorbance spectra of the same films. No significant changes in absorbance spectra of ZnO, S-ZnO (250 °C) and S-ZnO (350 °C) are observed, while the absorption of the S-ZnO annealed at 450 °C is higher, in accordance with the transmittance measurements. Moreover, the Tauc plots presented in Figure 4b, as derived from the UV-Vis absorption spectra, offer valuable insights into the direct optical bandgap of ZnO and S-ZnO films. The direct optical bandgap (E_g_) of the prepared samples was determined by extrapolating the linear section of each plot until they intersect the horizontal hv axis at (αhv)^2^ = 0. The calculated values of the bandgap of the pristine and S-doped ZnO films annealed at various temperatures are summarized in Table S4. The pristine ZnO film exhibits a direct bandgap of approximately 3.45 eV, which is consistent with its intrinsic properties. Interestingly, the S-ZnO annealed at 250 °C has the same optical bandgap, while the E_g_ values of the S-ZnO films at 350 °C and 450 °C are lower, with values of 3.38 and 3.22 eV, respectively. This bandgap modification at higher annealing temperatures may be indicative of the introduction–activation of intermediate energy levels upon sulfur doping, which occupy either substitutional or interstitial sites in the ZnO lattice. These intermediate states effectively narrow the bandgap, confirming the role of sulfur in modifying the electronic structure of ZnO. However, the excess of the intermediate states upon sulfurization at higher annealing temperatures could lead to charge losses, as they may act as traps for the photogenerated charges, when S-ZnO films annealed at 350 °C and 450 °C are introduced as electron extraction layers in inverted organic solar cells.

Moreover, the sulfur incorporation affected the optical disorder of the ZnO, which is attributed to the localized tail states. The tail of the density of electronic states that extend through the energy gap is referred to as the Urbach tail. The Urbach energy, which is the energy related to the Urbach tail, was calculated using Equation (8):

(8) $$a=a_{o}+\exp\left(\frac{E}{E_{u}}\right)$$

where *α* is the absorption coefficient, *E* is the photon energy and *E_u_* is the Urbach energy. By plotting the ln(*α*) versus the photon energy (Figure S3), the Urbach energy presented in Table S4 was estimated by taking the reciprocal of the slope of the linear fit. A slight improvement is observed between the pristine and S-doped ZnO annealed at 250 °C, while by increasing the annealing temperature, the *E_u_* enhances. This could be ascribed to the increase in the oxygen (O) vacancies due to the S-doping of the ZnO, leading to narrow bandgap values. O vacancies could improve the conductivity of S-doped films; nevertheless, the excessive number of defects may act as charge carrier traps, degrading the performance of OSCs using S-ZnO films as EELs.

**Figure 5:**
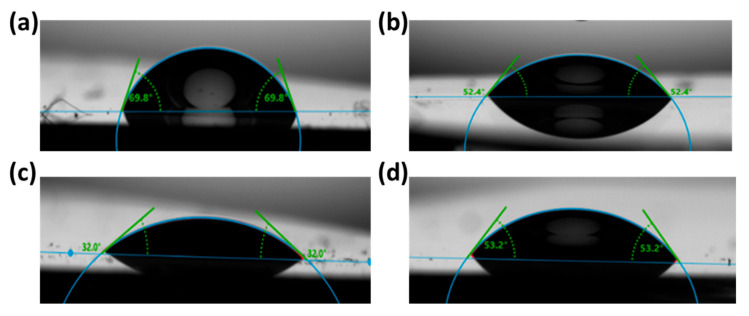
Water contact angle measurements taken on (**a**) ZnO and S-doped ZnO films annealed at (**b**) 250 °C (**c**) 350 °C and (**d**) 450 °C for 30 min.

Next, contact angle measurements were conducted to investigate the effect of the sulfur treatment on the surface properties of ZnO films. Note that according to the literature, the water contact angle of the ZnO film can vary from small to relatively large values, due to its tunable hydrophobicity, depending upon the synthesis method, deposition technique, surface crystal structure and surface roughness [72]. In general, ZnO surfaces are hydrophilic, characterized by a water contact angle of less than 90°. Figure 5a–d shows the wetting behavior of the ZnO and S-ZnO films, annealed at various temperatures. Sulfur-annealing at 250 °C and 350 °C enhances the hydrophilic nature of ZnO, as verified by the reduced water contact angle, from 69.8° for ZnO to 52.4° and 32°, respectively, for those two annealing temperatures, with 350 °C resulting in the most pronounced effect. On the other hand, annealing at 450 °C results in a less hydrophilic surface compared to annealing at 350 °C. However, it still remains more hydrophilic than the untreated ZnO.

**Figure 6:**
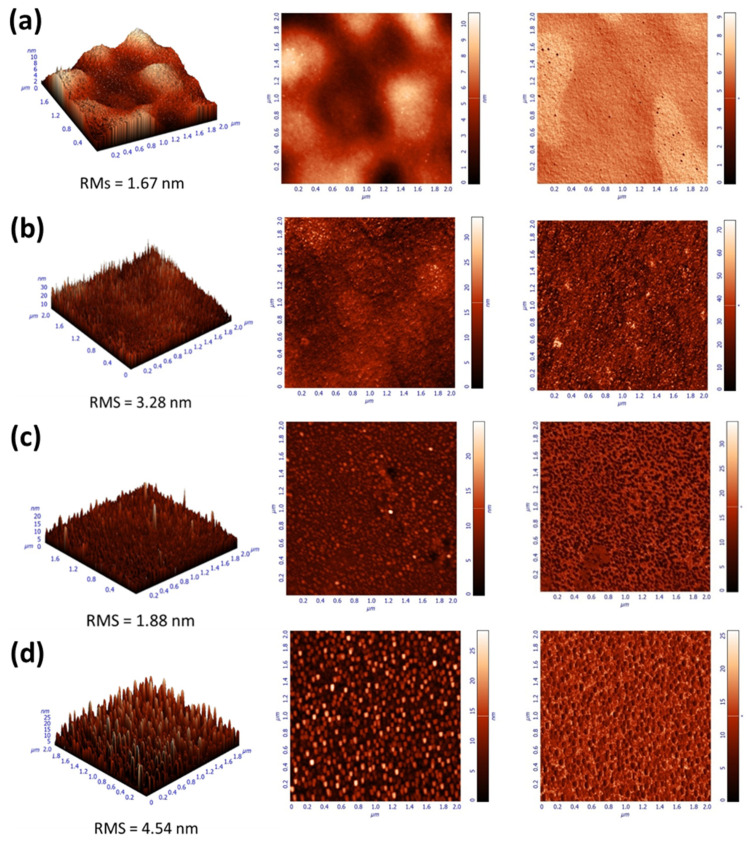
The 2 × 2 μm^2^ atomic force microscopy (AFM) images, 3D height (**left**), 2D height (**middle**) and phase (**right**), along with surface roughness measurements taken on the (**a**) pristine ZnO and S-doped ZnO annealed at (**b**) 250 °C, (**c**) 350 °C and (**d**) 450 °C.

The surface morphology of ZnO and S-ZnO films was further investigated by atomic force microscopy (AFM), and the resulting images are shown in Figure 6. The AFM results were correlated with the water contact angle measurements to elucidate the effects of the surface roughness and structural changes on wettability. Figure 6 shows the 2 × 2 μm^2^ AFM images of the pristine and sulfur-doped ZnO samples, where the left image refers to the 3D height, the middle refers to 2D height and the right to the phase image. As it can be seen in Figure 6a, the untreated ZnO surface exhibited the lowest roughness with an RMS value of 1.67 nm, indicative of a relatively smooth and uniform surface, with a small-sized grain (Figure S4a). This smooth morphology corresponds to a moderate water contact angle of 69.8°, reflecting the inherent hydrophobic nature of ZnO. Sulfur annealing at 250 °C (Figure 6b) led to a significant increase in roughness, with an RMS value of 3.28 nm, suggesting surface restructuring and the development of larger grains (Figure S4b). The increased roughness enhanced the water spreading, resulting in a reduced contact angle of 52.4°. This behavior aligns with the Wenzel model [73], according to which the increased roughness on a hydrophilic surface enhances wettability by allowing greater interaction between the water and the surface. At 350 °C (Figure 6c), the roughness decreased to 1.88 nm, indicating the partial smoothing of the surface. However, the size of the grains enhanced (Figure S4c). Despite the reduction in roughness, the water contact angle decreased further to 32°, likely due to the sulfur incorporation and chemical modification of the surface. This suggests that the surface chemistry, in addition to roughness, plays a dominant role in enhancing hydrophilicity at this temperature. Annealing at 450 °C (Figure 6d) resulted in a significant increase in surface roughness, with an RMS value of 4.54 nm, indicative of grain growth or the formation of larger surface structures (Figure S4d). The corresponding contact angle increased to 53.2°, suggesting a reduction in hydrophilicity. This behavior may arise from the combined effects of the increased roughness creating mixed wetting regimes and possible changes in the surface chemistry, such as a reduction in polar functional groups.

Another critical factor is the surface energy of the pristine and S-doped ZnO films. It is already shown that sulfur doping can change the nanomorphology and wettability of the ZnO and, thus, could impact their surface energy. Therefore, the surface energy (Table S5) of all samples was estimated using the contact angle measurements of the droplets of the polar (i.e., water), as already presented in Figure 5, and non-polar (i.e., diiodomethane) components, as shown in Figure S5. The surface energy of the ZnO and S-ZnO annealed at the moderate temperature of 250 °C is 45.51 and 53.01 mJ m^−2^, while upon increasing the annealing temperature during the sulfurization process the surface energy is higher— 67.86 and 55.82 mJ m^−2^ for the S-doped ZnO annealed at 350 °C and 450 °C. As is already presented [74], a moderate surface energy of around 50 mJ m^−2^ is beneficial for the good interfacial contact between the oxide and the photoactive layer of an OSC. It is evident that the S-ZnO annealed at 250 °C is a good candidate for an efficient EEL in inverted OSCs, improving the hydrophilicity of ZnO and achieving desirable surface energy that could lead to a better adhesion of the PCE10:IT-4F photoactive layer deposited on top.

**Figure 7:**
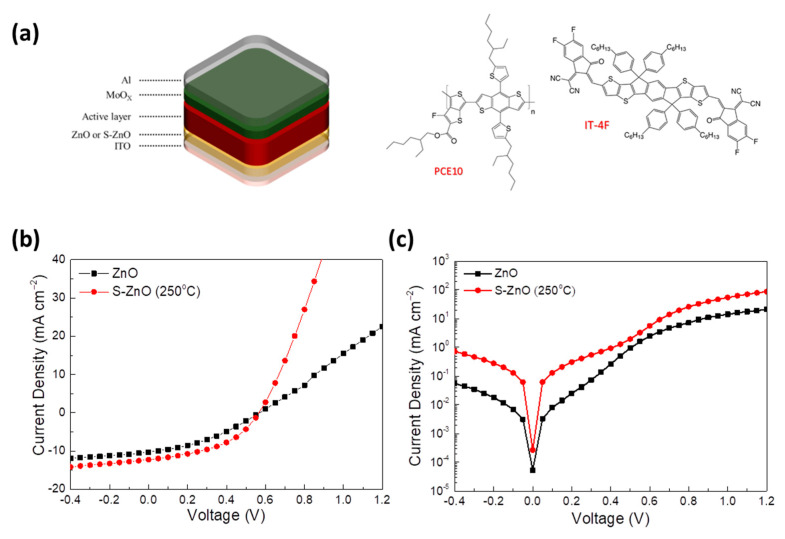
(**a**) Schematic illustration of inverted organic solar cell architecture and chemical structures of organic semiconductors used in this study. (**b**) Current density–voltage (J–V) characteristic curves of fabricated inverted OSCs using PCE10:IT-4F as photoactive layer and either ZnO or S-ZnO EELs annealed at 250 °C upon 1.5 AM irradiation. (**c**) Dark J–V characteristics of same devices.

To find out whether the sulfur-treated ZnO annealed at various temperatures has a positive impact on the device performance, S-ZnO films were applied as cathode interlayers in inverted organic solar cells (OSCs). The inverted device architecture and the molecular structures of the organic semiconductors used in this study are shown in Figure 7a. The current density–voltage (J–V) curves of the PCE10:IT-4F-based organic solar cells (OSCs) were measured under simulated 1.5 AM solar irradiation. In the OSCs, ZnO or sulfur-doped ZnO (S-ZnO) materials were used as electron extraction interlayers, annealed at varying temperatures for 30 min. Figure 7b shows the experimental J–V curves, while Table 1 summarizes the corresponding electrical performance parameters. The dark J–V measurements of the same devices are depicted in Figure 7c. From the J–V curves shown in Figure 7b, it becomes evident that, compared to the reference device with the pristine ZnO, the device using the S-ZnO annealed at 250 °C exhibits improved operational characteristics. On the other hand, at higher annealing temperatures of 350 °C and 450 °C, the device’s operational characteristics completely deteriorate, as revealed from the J–V characteristic curves presented in Figure S6. According to the photovoltaic parameters of Table 1, the reference device with the pristine ZnO exhibited a short circuit current density (J_sc_) of 10.28 mA cm^−2^, an open circuit voltage (V_oc_) of 0.57 V and a fill factor of 0.36, yielding a PCE of 2.11%. After doping the ZnO with sulfur and annealing at 250 °C, although no change in the V_oc_ value was observed, a significant enhancement in the J_sc_ (12.26 mA cm^−2^) and FF (0.45) is obtained, thus increasing the PCE to 3.14%, with an efficiency improvement of 49% compared to the reference device. In order to explain the significant enhancement of the device performance, the series (R_s_) and shunt (R_sh_) resistance of the aforementioned devices were calculated from the J–V characteristics (also listed in Table 1). It is well established that the R_s_ represents the sum of resistances that occur across the solar cell junctions due to ohmic losses. This involves the contact resistance at the various interfaces as well as the bulk resistance of all the deposited layers within the cell. On the other hand, the R_sh_ is associated with the charge carrier losses eventuating from charge recombination and current leakage pathways in the bulk or at the different interfaces of the device. The series resistance of the device incorporating the S-ZnO interlayer annealed at 250 °C is considerably decreased from 26.34 for the reference device with the pristine ZnO to 6.40 Ω cm^2^, suggesting a considerably improved film conductivity and/or a more efficient electron transfer/collection leading to the enhancement of the J_sc_ and FF. Moreover, the marginally increased value of the shunt resistance of the device, from 162.88 to 177.54 Ω cm^2^, is consistent with the unchanged V_oc_ value upon S doping. The significantly reduced R_s_ and the small increase in the R_sh_ are consistent with the enhanced dark current observed at positive voltages (Figure 7c) for the device with the 250 °C-annealed S-doped ZnO layer. These findings indicate an improved interfacial contact between the active layer and the modified cathode and/or enhanced electron transport in the S-doped ZnO (250 °C), which facilitates the electron transfer/extraction and is probably due to the enhanced (n-type) ZnO conductivity/mobility, owing to the incorporation of sulfur atoms in the ZnO lattice. This improved n-type conductivity upon S-doping is also confirmed by J–V characteristic curves taken in ITO/ZnO/Al and ITO/S-ZnO (250 °C)/Al devices, shown in Figure S7. A better ohmic contact is observed upon the sulfurization of ZnO, which is beneficial for the improvement of the electron extraction of the inverted OSC. Note that the absorbance measurements on PCE10:IT-4F films deposited on both the untreated and the sulfur-treated ZnO layer showed no appreciable change (Figure S8), suggesting that the nanomorphology of the active layer on top of the pristine or the S-doped ZnO layer is unlikely to be the main factor contributing to the significant photocurrent enhancement of the device with the sulfurized EEL.

**Figure 8:**
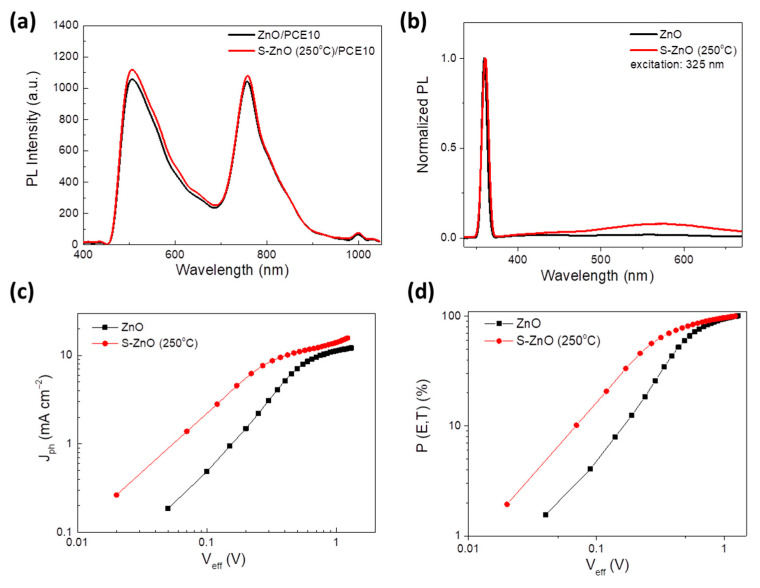
(**a**) Steady state photoluminescence (PL) spectra of PCE10 deposited on top of ZnO and S-ZnO annealed at 250 °C. (**b**) Steady state PL spectra of ZnO and S-ZnO (250 °C) films deposited on glass substrates. (**c**) Net photocurrent density and (**d**) exciton dissociation probability (*P*(*E*,*T*)) plotted versus effective voltage of OSCs using either ZnO or S-ZnO (250 °C) as EELs.

In order to investigate the origin of the improved device performance using the S-ZnO annealed at a moderate temperature of 250 °C as the EEL, the photophysical properties of the donor PCE10 deposited on the pristine and S-doped ZnO were measured. Steady state photoluminescence (PL) measurements on the PCE10 deposited on the pristine ZnO and S-ZnO (250 °C) are shown in Figure 8a. Both emission peaks observed at around 520 nm and 770 nm reflect the typical photophysical behavior of PCE10, where excitons are generated and recombine radiatively. The small redshift of the PL spectra of PCE10 in the sulfurized ZnO case may be attributed to the decreased non-radiative interfacial exciton recombination as a result of the surface passivation effect of the sulfur treatment which also led to a small enhancement of the PL, without any modification of its spectral characteristics.

Figure 8b presents the PL spectra of ZnO and S-ZnO films annealed at 250 °C, both normalized at the near-band-edge (NBE) peak, highlighting the impact of the sulfur doping and thermal treatment on their optical characteristics. No significant change in the NBE emission of the ZnO and S-ZnO (250 °C) films at 359 nm (3.45 eV) and 360 nm (3.44 eV), respectively, is observed. On the other hand, the incorporation of S in the ZnO lattice induces sulfur-related defects (like sulfur interstitials or substitutional sulfur) and vacancies, due to which a green emission band near 570 nm is observed. A minor redshift in the PL spectrum of the S-ZnO, as well as the small enhancement of the overall PL signal, is observed compared to the ZnO PL spectrum, suggesting the existence of a sulfur emission center as well as the slight increase in the number of O vacancies leading to the increased conductivity of the S-ZnO film [75]. On the other hand, the excessive S-doping upon sulfurization at 350 °C and 450 °C results in narrowing the bandgap of the ZnO, as revealed from the Tauc plots, which favors the photocatalytic activity of ZnO. However, the photocatalytic activity significantly affects the non-fullerene acceptor, IT-4F [76,77], resulting in the poor PCE of the devices based on the S-ZnO annealed at 350 °C and 450 °C, as is evident from the J–V characteristic curves (Figure S6). The poor performance of the devices based on S-ZnO layers annealed at 350 °C and 450 °C could also be attributed to charge losses due to the excess of intermediate states created upon sulfurization at high temperatures, as verified by absorbance measurements and Tauc plots (Figure 4b and Figure S2), acting as traps of the photogenerated charges. Therefore, we can conclude that in the case of the S-ZnO annealed at 250 °C, sulfur improves the conductivity and also increases the crystalline size of the ZnO, leading to an improved electron extraction and thus an enhanced device performance when it is introduced in inverted OSCs.

Moreover, the nanomorphology of the photoactive layer deposited on top of the pristine and S-doped ZnO was investigated. The 2 × 2 μm^2^ AFM images of the PCE10:IT-4F spin-coated on the ZnO and S-ZnO (250 °C) are presented in Figure S9a,b, respectively. A significant reduction in the RMS roughness of the photoactive layer deposited on the S-ZnO (250 °C) (RMS of 0.38 nm), as compared with that spin-coated on the pristine ZnO (RMS of 0.57 nm), was estimated, suggesting a better PCE10:IT-4F film formation. This improved physical contact between the S-ZnO (250 °C) EEL and the photoactive layer resulted in a better electrical contact and, thus, enhanced the electron transport and extraction. The nanomorphology of the PCE10:IT-4F deposited on the S-ZnO (350 °C) and S-ZnO (450 °C) was also investigated for comparison reasons (Figure S9c,d, respectively). As the annealing temperature of the S-ZnO increased, the RMS roughness of the photoactive film increased. This rough surface and the uneven surface could lead to the poor electrical contact of the S-ZnO (350 °C)/PCE10:IT-4F and S-ZnO (450 °C) and, thus, poor device performance.

Finally, in order to investigate the effect of the sulfurization of the ZnO on the device photocurrent properties, the dependence of the (net) photocurrent density, *J_ph_*, versus the effective voltage, *V_eff_*, for the devices incorporating ZnO and S-ZnO (250 °C) interlayers was examined (Figure 8c).The *J_ph_* is determined via the following formula (Equation (9)):

*J_ph_* = *J_L_* − *J_D_* (9)

where *J_L_* and *J_D_* represent the current density under illumination and in the dark, respectively. The *V_eff_* is acquired with Equation (10):

*V_eff_* = *V*_0_ − *V* (10)

where *V*_0_ stands for the voltage when the *J_ph_* equals zero and *V_appl_* is the applied voltage. Evidently, the *J_ph_* is higher in the S-ZnO-based cell and reaches saturation (*J_ph,sat_*) at the lower *V_eff_* in respect to the reference device using the ZnO layer, suggesting that sulfur doping is beneficial for the exciton dissociation into free carriers. Consequently, the maximum exciton generation rate, *G_max_*, is higher in the S-ZnO-based cell compared to the device based on the pristine ZnO layer, since the *G_max_* is proportional to the *J_ph,sat_*. In addition, at a low *V_eff_* (<0.6 V), the exciton dissociation probability, estimated by Equation (11), of the S-ZnO-based device is higher than the cell with the ZnO, as shown in Figure 8d.

(11) $$P(E,T)=\frac{J_{ph}}{J_{ph,sat}}\cdot 100\%$$

In particular, under short-circuit conditions the S-ZnO OSC exhibits a high *P*(*E*,*T*) of 90%, while the reference device shows a *P*(*E*,*T*) of 86%. This result, in combination with the previous ones, suggests that the sulfur-doped ZnO annealed at moderate temperatures is more efficient in transporting and extracting electrons as well as in enhancing the exciton dissociation and device photocurrent generation than its as-deposited counterpart, thus resulting in an increased PCE in inverted OSCs with doped ZnO-modified cathode electrodes.

## 4. Conclusions

To summarize, the effectiveness of sulfur doping in enhancing the performance of zinc oxide (ZnO) electron extraction layers (EELs) in inverted organic solar cells (OSCs) was demonstrated. Sulfur treatment at a moderate annealing temperature (250 °C) resulted in a 49% improvement in power conversion efficiency (PCE), increasing it from 2.11% for the reference device with pristine ZnO to 3.14% for the OSC based on the S-ZnO (250 °C). This enhancement was primarily attributed to the improved short-circuit current density (J_sc_) and fill factor (FF), likely stemming from the enhanced electron transport capability of the S-doped ZnO film. Structural and optical analyses revealed that the sulfur incorporation in the ZnO at a moderate annealing temperature did not significantly affect its optical bandgap but led to a small increase in the crystallite size and likely enhanced its n-type conductivity, thus facilitating a more efficient electron transport/extraction, interfacial exciton dissociation and charge generation in inverted OSCs. The minimal impact of sulfurization on the ZnO film morphology ensures the compatibility of this approach with existing OSC fabrication processes, especially in an inverted device architecture. These findings suggest that sulfur doping offers a simple and scalable strategy to improve the charge-transport properties and PCE of ZnO-based inverted OSCs, paving the way for further advancements in inverted OSC architectures with doped metal oxide-modified cathode electrodes.

## Figures and Tables

**Table 1:** Performance parameters of OSCs with the inverted structure of the ITO/ZnO or S-ZnO/PCE10:IT-4F/MoO_X_/Al.

EEL	J_sc_ (mA cm^−2^)	V_oc_ (V)	FF	PCE (%)	R_s_ (Ω cm^2^)	R_sh_ (Ω cm^2^)
ZnO	10.28	0.57	0.36	2.11	26.34	162.88
S-ZnO (250 °C)	12.26	0.57	0.45	3.14	6.40	177.54

## Data Availability

The original contributions presented in this study are included in the article/Supplementary Materials. Further inquiries can be directed to the corresponding authors.

## Supplementary Materials

The following supporting information can be downloaded at: https://www.mdpi.com/article/10.3390/ma18081767/s1, Figure S1: (a) FTIR transmittance spectra and (b) enlarged area of the same spectra of pristine and sulfurized ZnO annealed at 250 °C for 30 min. Figure S2: UV-Vis absorption spectra of pristine and sulfurized ZnO thin films annealed at various temperatures for 30 min. Figure S3: Urbach energy of pristine and sulfurized ZnO thin films annealed at various temperatures for 30 min. Figure S4: Histogram of grain size of (a) ZnO, (b) S-ZnO (250 °C), (c) S-ZnO (350 °C) and (d) S-ZnO (450 °C) substrates. Figure S5: Contact angle measurements of a droplet of diiodomethane on ZnO and S-ZnO substrates annealed at various temperatures for 30 min. Figure S6: Current density–voltage (J–V) characteristic curves of inverted OSCs based on S-ZnO EELs annealed at 350 °C and 450 °C for 30 min. Figure S7: J–V curves of the devices with the architecture ITO/ZnO or S-ZnO/Al. Figure S8: UV-Vis absorption spectra of PCE10:IT-4F spin-coated on pristine and sulfurized ZnO thin films annealed at 250 °C for 30 min. Figure S9: 2 × 2 μm^2^ AFM height images of PCE10:IT-4F spin-coated on (a) ZnO, (b) S-ZnO (250 °C), (c) S-ZnO (350 °C), and (d) S-ZnO (450 °C) substrates. Table S1: XRD analysis showing d-spacing and crystalline size (D) of ZnO and S-ZnO annealed at 250 °C for 30 min. Table S2: XRD analysis showing lattice parameters (α and c), unit cell volume (V), and bond length (L) of ZnO and S-ZnO annealed at 250 °C for 30 min. Table S3: Energy dispersive X-ray spectroscopy (EDX) analysis for pristine and S-doped ZnO samples annealed at various temperatures. Table S4: Calculated values of band gap and Urbach energy of pristine and S-doped ZnO films annealed at various temperatures. Table S5: Surface energy as derived from contact angle measurements of ZnO and S-ZnO substrates.

## References

[1] Lewis N.S. (2007). Toward Cost-Effective Solar Energy Use. Science.

[2] An Q., Wang J., Zhang F. (2019). Ternary polymer solar cells with alloyed donor achieving 14.13% efficiency and 78.4% fill factor. Nano Energy.

[3] Hu Z., Wang Z., An Q., Zhang F. (2020). Semitransparent polymer solar cells with 12.37% efficiency and 18.6% average visible transmittance. Sci. Bull..

[4] Wang X., Sun Q., Gao J., Wang J., Xu C., Ma X., Zhang F. (2021). Recent progress of organic photovoltaics with efficiency over 17%. Energies.

[5] Han S., Deng Y., Han W., Ren G., Song Z., Liu C., Guo W. (2021). Recent advances of semitransparent organic solar cells. Sol. Energy.

[6] Chen H., Huang Y., Zhang R., Mou H., Ding J., Zhou J., Wang Z., Li H., Chen W., Zhu J. (2025). Organic solar cells with 20.82% efficiency and high tolerance of active layer thickness through crystallization sequence manipulation. Nat. Mater..

[7] Cui Y., Yao H., Zhang J., Xian K., Zhang T., Hong L., Wang Y., Xu Y., Ma K., An C. (2020). Single-junction organic photovoltaic cells with approaching 18% efficiency. Adv. Mater..

[8] Cui Y., Yao H., Hong L., Zhang T., Tang Y., Lin B., Xian K., Gao B., An C., Bi P. (2019). 17% efficiency organic photovoltaic cell with superior processability. Natl. Sci. Rev..

[9] Li C., Zhou J., Song J., Xu J., Zhang H., Zhang X., Guo J., Zhu L., Wei D., Han G. (2021). Non-fullerene acceptors with branched side chains and improved molecular packing to exceed 18% efficiency in organic solar cells. Nat. Energy.

[10] Zhang Z., Li Y., Cai G., Zhang Y., Lu X., Lin Y. (2020). Selenium heterocyclic electron acceptor with small urbach energy for as-cast high-performance organic solar cells. J. Am. Chem. Soc..

[11] Liu Q., Jiang Y., Jin K., Qin J., Xu J., Li W., Xiong J., Liu J., Xiao Z., Sun K. (2020). 18% efficiency organic solar cells. Sci. Bull..

[12] Wang T., Sun R., Shi M., Pan F., Hu Z., Huang F., Li Y., Min J. (2020). Solution-processed polymer solar cells with over 17% efficiency enabled by an iridium complexation approach. Adv. Energy Mater..

[13] Wu J., Li G., Fang J., Guo X., Zhu L., Guo B., Wang Y., Zhang G., Arunagiri L., Liu F. (2020). Random terpolymer based on thiophene-thiazolothiazole unit enabling efficient non-fullerene organic solar cells. Nat. Commun..

[14] Ma R., Liu T., Luo Z., Guo Q., Xiao Y., Chen Y., Li X., Luo S., Lu X., Zhang M. (2020). Improving open-circuit voltage by a chlorinated polymer donor endows binary organic solar cells efficiencies over 17%. Sci. China Chem..

[15] Yao J., Qiu B., Zhang Z.-G., Xue L., Wang R., Zhang C., Chen S., Zhou Q., Sun C., Yang C. (2020). Cathode engineering with perylene-diimide interlayer enabling over 17% efficiency single-junction organic solar cells. Nat. Commun..

[16] Kang Q., Zheng Z., Zu Y., Liao Q., Bi P., Zhang S., Yang Y., Xu B., Hou J. (2021). n-doped inorganic molecular clusters as a new type of hole transport material for efficient organic solar cells. Joule.

[17] Lin Y., Adilbekova B., Firdaus Y., Yengel E., Faber H., Sajjad M., Zheng X., Yarali E., Seitkhan A., Bakr O.M. (2019). 17% efficient organic solar cells based on liquid exfoliated WS_2_ as a replacement for PEDOT:PSS. Adv. Mater..

[18] Yu G., Gao J., Hummelen J.C., Wudl F., Heeger A.J. (1995). Polymer photovoltaic cells: Enhanced efficiencies via a network of internal donor-acceptor heterojunctions. Science.

[19] Günes S., Neugebauer H., Sariciftci N.S. (2007). Conjugated polymer-based organic solar cells. Chem. Rev..

[20] Dennler G., Scharber M.C., Brabec C.J. (2009). Polymer-fullerene bulk-heterojunction solar cells. Adv. Mater..

[21] Li G., Zhu R., Yang Y. (2012). Polymer solar cells. Nat. Photonics.

[22] Kim Y.H., Lee S.H., Noh J., Han S.H. (2006). Performance and stability of electroluminescent device with self-assembled layers of poly(3,4-ethylenedioxythiophene)-poly(styrenesulfonate) and polyelectrolytes. Thin Solid Film..

[23] De Jong M.P., van IJzendoorn L.J., de Voigt M.J.A. (2000). Stability of the interface between indium-tin-oxide and poly(3,4-ethylenedioxythiophene)/poly(styrenesulfonate) in polymer light-emitting diodes. Appl. Phys. Lett..

[24] Kemerink M., Timpanaro S., De Kok M.M., Meulenkamp E.A., Touwslager F.J. (2004). Three-dimensional inhomogeneities in PEDOT:PSS films. J. Phys. Chem. B.

[25] Lu L., Zheng T., Wu Q., Schneider A.M., Zhao D., Yu L. (2015). Recent advances in bulk heterojunction polymer solar cells. Chem. Rev..

[26] Wang K., Liu C., Meng T., Yi C., Gong X. (2016). Inverted organic photovoltaic cells. Chem. Soc. Rev..

[27] Lattante S. (2014). Electron and hole transport layers: Their use in inverted bulk heterojunction polymer solar cells. Electronics.

[28] Dahiya H., Suthar R., Khandelwal K., Karak S., Sharma G.D. (2022). Recent advances in organic and inorganic hole and electron transport layers for organic solar cells: Basic concept and device performance. ACS Appl. Electron. Mater..

[29] Li T., Chen Z., Wang Y., Tu J., Deng X., Li Q., Li Z. (2019). Materials for interfaces in organic solar cells and photodetectors. ACS Appl. Mater. Interfaces.

[30] Hewlett R.M., McLachlan M.A. (2016). Surface structure modification of ZnO and the impact on electronic properties. Adv. Mater..

[31] Kamalasanan M.N., Chandra S. (1996). Sol-gel synthesis of ZnO thin films. Thin Solid Film..

[32] Han Y., Guo J., Luo Q., Ma C.-Q. (2023). Solution-processable zinc oxide for printed photovoltaics: Progress, challenges, and prospect. Adv. Energy Sustain. Res..

[33] Kohan A.F., Ceder G., Morgan D., Van de Walle C.G. (2000). First-principles study of native point defects in ZnO. Phys. Rev. B.

[34] Janotti A., Van de Walle C.G. (2005). Oxygen vacancies in ZnO. Appl. Phys. Lett..

[35] Janotti A., Van de Walle C.G. (2007). Native point defects in ZnO. Phys. Rev. B.

[36] Ha Y.E., Jo M.Y., Park J., Kang Y.-C., Yoo S.I., Kim J.H. (2013). Inverted type polymer solar cells with self-assembled monolayer treated ZnO. J. Phys. Chem. C.

[37] Ha Y.E., Jo M.Y., Park J., Kang Y.-C., Moon S.-J., Kim J.H. (2014). Effect of self-assembled monolayer treated ZnO as an electron transporting layer on the photovoltaic properties of inverted type polymer solar cells. Synth. Met..

[38] Sin D.H., Kim S.H., Lee J., Lee H. (2022). Modification of electrode interface with fullerene-based self-assembled monolayer for high-performance organic optoelectronic devices. Micromachines.

[39] Gebremariam K.G., Hone F.G., Negash A., Genene Z., Dai J., Waketola A.G., Mola G.T., Mammo W., Tegegne N.A. (2024). Self-assembled monolayer engineered ZnO electron transport layer to improve the photostability of organic solar cells. Energy Fuels.

[40] Hu L., Chen L., Hu X., Chen Y. (2014). Solution processed and self-assembled polymerizable fullerenes/metal oxide as an interlayer for high efficient inverted polymer solar cells. J. Mater. Chem. C.

[41] Tountas M., Topal Y., Verykios A., Soultati A., Kaltzoglou A., Papadopoulos T.A., Auras F., Seintis K., Fakis M., Palilis L.C. (2018). A silanol-functionalized polyoxometalate with excellent electron transfer mediating behavior to ZnO and TiO_2_ cathode interlayers for highly efficient and extremely stable polymer solar cells. J. Mater. Chem. C.

[42] Seo J.H., Gutacker A., Sun Y.M., Wu H.B., Huang F., Cao Y., Scherf U., Heeger A.J., Bazan G.C. (2011). Improved high-efficiency organic solar cells via incorporation of a conjugated polyelectrolyte interlayer. J. Am. Chem. Soc..

[43] Jin W.-Y., Ginting R.T., Jin S.-H., Kang J.-W. (2016). Highly stable and efficient inverted organic solar cells based on low-temperature solution-processed PEIE and ZnO bilayers. J. Mater. Chem. A.

[44] You H., Zhang J., Zhang Z., Zhang C., Lin Z., Chang J., Han G., Zhang J., Lu G., Hao Y. (2017). Low temperature aqueous solution-processed ZnO and polyethylenimine ethoxylated cathode buffer bilayer for high performance flexible inverted organic solar cells. Energies.

[45] He Z., Zhong C., Su S., Xu M., Wu H., Cao Y. (2012). Enhanced power-conversion efficiency in polymersolar cells using an inverted device structure. Nat. Photonics.

[46] Zhou Y., Fuentes-Hernandez C., Shim J., Meyer J., Giordano A., Li H., Winget P., Papadopoulos T.A., Cheun H., Kim J. (2012). A universal method to produce low-work function electrodes for organic electronics. Science.

[47] Lee B.R., Lee W., Nguyen T.L., Park J.S., Kim J.-S., Kim J.Y., Woo H.Y., Song M.H. (2013). Highly efficient red-emitting hybrid polymer light-emitting diodes via Förster resonance energy transfer based on homogeneous polymer blends with the same polyfluorene backbone. ACS Appl. Mater. Interfaces.

[48] Lee B.R., Choi H., SunPark J., Lee H.J., Kim S.O., Kim J.Y., Song M.H. (2011). Surface modification of metal oxide using ionic liquid molecules in hybrid organic–inorganic optoelectronic devices. J. Mater. Chem..

[49] Zhang X., Cui M., Nian L., Wang P., Rong Q., Shui L., Coehoorn R., Zhou G., Li N. (2020). Ionic liquid-modified ZnO-based electron transport layer for inverted organic solar cells. J. Mater. Sci. Mater. Electron..

[50] Polydorou E., Zeniou A., Tsikritzis D., Soultati A., Sakellis I., Gardelis S., Papadopoulos T.A., Briscoe J., Palilis L.C., Kennou S. (2016). Surface passivation effect by fluorine plasma treatment on ZnO for efficiency and lifetime improvement of inverted polymer solar cells. J. Mater. Chem. A.

[51] Papamakarios V., Polydorou E., Soultati A., Droseros N., Tsikritzis D., Douvas A.M., Palilis L., Fakis M., Kennou S., Argitis P. (2016). Surface modification of ZnO layers via hydrogen plasma treatment for efficient inverted polymer solar cells. ACS Appl. Mater. Interfaces.

[52] Polydorou E., Botzakaki M.A., Sakellis I., Soultati A., Kaltzoglou A., Papadopoulos T.A., Briscoe J., Drivas C., Seintis K., Fakis M. (2017). Improved stability of polymer solar cells in ambient air via atomic layer deposition of ultrathin dielectric layers. Adv. Mater. Interfaces.

[53] Polydorou E., Botzakaki M., Drivas C., Seintis K., Sakellis I., Soultati A., Kaltzoglou A., Speliotis T., Fakis M., Palilis L.C. (2018). Insights into the passivation effect of atomic layer deposited hafnium oxide for efficiency and stability enhancement in organic solar cells. J. Mater. Chem. C.

[54] Lilliedal M.R., Medford A.J., Madsen M.V., Norrman K., Krebs F.C. (2010). The effect of post-processing treatments on inflection points in current-voltage curves of roll-to-roll processed polymer photovoltaics. Sol. Energy Mater. Sol. Cells.

[55] Bao Q., Liu X., Xia Y., Gao F., Kauffmann L.-D., Margeat O., Ackermann J., Fahlman M. (2014). Effects of ultraviolet soaking on surface electronic structures of solution processed ZnO nanoparticle films in polymer solar cells. J. Mater. Chem. A.

[56] Lee H.B., Ginting R.T., Tan S.T., Tan C.H., Alshanableh A., Oleiwi H.F., Yap C.C., Jumali M.H.H., Yahaya M. (2016). Controlled defetcs of fluorine-incorporated ZnO nanorods for photovoltaic enhancement. Sci. Rep..

[57] Soultati A., Fakharuddin A., Polydorou E., Drivas C., Kaltzoglou A., Haider M.I., Kournoutas F., Fakis M., Palilis L.C., Kennou S. (2019). Lithium doping of ZnO for high efficiency and stability fullerene and non-fullerene organic solar cells. ACS App. Energy Mater..

[58] Wang J., Pan H., Xu X., Jin H., Ma W., Xiong S., Bao Q., Tang Z., Ma Z. (2022). Li-doped ZnO electron transport layer for improved performance and photostability of organic solar cells. ACS Appl. Mater. Interfaces.

[59] Ierides I., Ligorio G., McLachlan M.A., Guo K., List-Kratochvil E.J.W., Cacialli F. (2022). Inverted organic photovoltaics with a solution-processed Mg-doped ZnO electron transport layer annealed at 150 °C. Sustain. Energy Fuels.

[60] Pachoumi O., Li C., Vaynzof Y., Banger K.K., Sirringhaus H. (2013). Improved performance and stability of inverted organic solar cells with sol–gel processed, amorphous mixed metal oxide electron extraction layers comprising alkaline earth metals. Adv. Energy Mater..

[61] Shin K.-S., Lee K.-H., Lee H.H., Choi D., Kim S.-W. (2010). Enhanced power conversion efficiency of inverted organic solar cells with a Ga-doped ZnO nanostructured thin film prepared using aqueous solution. J. Phys. Chem. C.

[62] Zhang Q., Peng R., Zhang C., Chen D., Lin Z., Chang J., Zhang J., Hao Y. (2018). Inverted organic solar cells with low-temperature Al-doped-ZnO electron transport layer processed from aqueous solution. Polymers.

[63] Liu X., Ji Y., Xia Z., Zhang D., Cheng Y., Liu X., Ren X., Liu X., Huang H., Zhu Y. (2024). In-doped ZnO electron transport layer for high-efficiency ultrathin flexible organic solar cells. Adv. Sci..

[64] Polydorou E., Soultati A., Vasilopoulou M. (2016). Highly conductive, optically transparent, low work-function hydrogen-doped boron-doped ZnO electrodes for efficient ITO-free polymer solar cells. J. Mater. Chem. C.

[65] Patil A.B., Patil K.R., Pardeshi S.K. (2010). Ecofriendly synthesis and solar photocatalytic activity of S-doped ZnO. J. Hazard. Mater..

[66] Wang X.H., Liu S., Chang P., Tang Y. (2007). Synthesis of sulfur-doped ZnO nanowires by electrochemical deposition. Mater. Sci. Semicond. Process..

[67] Zafar M., Yun J.-Y., Kim D.-H. (2019). Improved inverted-organic-solar-cell performance via sulfur doping of ZnO films as electron buffer layer. Mater. Sci. Semicond. Process..

[68] Lin M.-H., Ho C.-H. (2017). Synthesis and optical characterization of oxygen-incorporated ZnS_(1–x)_O_x_ for UV-visible color palette light-emission matter. ACS Omega.

[69] Bulliard X., Ihn S.-G., Yun S., Kim Y., Choi D., Choi J.-Y., Kim M., Sim M., Park J.-H., Choi W. (2010). Enhanced performance in polymer solar cells by surface energy control. Adv. Funct. Mater..

[70] Bindu P., Thomas S. (2014). Estimation of lattice strain in ZnO nanoparticles: X-ray peak profile analysis. J. Theor. Appl. Phys..

[71] Wenzel R.N. (1936). Resistance of solid surfaces to wetting by water. Ind. Eng. Chem. Res..

[72] Xu C.L., Fang L., Wu F., Huang Q.L., Yin B. (2014). Wetting behavior of triethoxyoctylsilane modified ZnO nanowire films. Colloids Surf. A Physicochem. Eng. Asp..

[73] Hsu C.-L., Su I.-L., Hsueh T.-J. (2015). Sulfur-doped-ZnO-nanospire-based transparent flexible nanogenerator self-powered by environmental vibration. RSC Adv..

[74] Wang Z., Yang Y., Zhang L., Lin H., Zhang Z., Wang D., Peng S., He D., Ye J., Gao P. (2018). Modulation-doped ZnO as high performance electron-selective layer for efficient silicon heterojunction solar cells. Nano Energy.

[75] Zhou P., Yu X., Yang L., Tao Z. (2007). Simple air oxidation synthesis and optical properties of S-doped ZnO microspheres. Mater. Lett..

[76] Jiang Y., Sun L., Jiang F., Xie C., Hu L., Dong X., Qin F., Liu T., Hu L., Jiang X. (2019). Photocatalytic effect of ZnO on nonfullerene acceptors and its mitigation by SnO_2_ for nonfullerene organic solar cells. Mater. Horiz..

[77] Soultati A., Verykios A., Panagiotakis S., Armadorou K.-K., Haider M.I., Kaltzoglou A., Drivas C., Fakharuddin A., Bao X., Yang C. (2020). Suppressing the photocatalytic activity of zinc oxide electron-transport layer in nonfullerene organic solar cells with a pyrene-bodipy interlayer. ACS Appl. Mater. Interfaces.

