# Supplementary material for: Sulfur-Doped ZnO as Cathode Interlayer for Efficient Inverted Organic Solar Cells
Source: Materials (Basel). 2025 Apr 12;18(8):1767. doi: 10.3390/ma18081767 (PMC12029029; doi:10.3390/ma18081767)
Supplement: Supplementary file 1 [file materials-18-01767-s001.zip › materials-3557762-supplementary.pdf]

Supporting Information

Sulfur-doped ZnO as Cathode Interlayer for Efficient Inverted Organic Solar Cells

Ermioni Polydorou[1,*], Georgios Manginas[2], Georgios Chatzigiannakis[1,3], Zoi Georgiopoulou[1,3], Apostolis Verykios[1], Elias Sakellis[1,3], Maria Eleni Rizou[1], Vassilis Psycharis[1], Leonidas Palilis[4], Dimitris Davazoglou[1], Anastasia Soultati[1,*], Maria Vasilopoulou[1,*].

[1]*Institute of Nanoscience and Nanotechnology (INN), National Center for Scientific Research (NCSR) Demokritos, 15341 Agia Paraskevi, Attica, Greece*

[2]*University of West Attica, 12244 Egaleo, Athens, Greece*

[3]*Solid State Physics Section, Department of Physics, National and Kapodistrian University of Athens, Panepistimioupolis, 15784 Zografos, Athens, Greece*

[4]*Department of Physics, University of Patras, Patras, 26504, Greece*

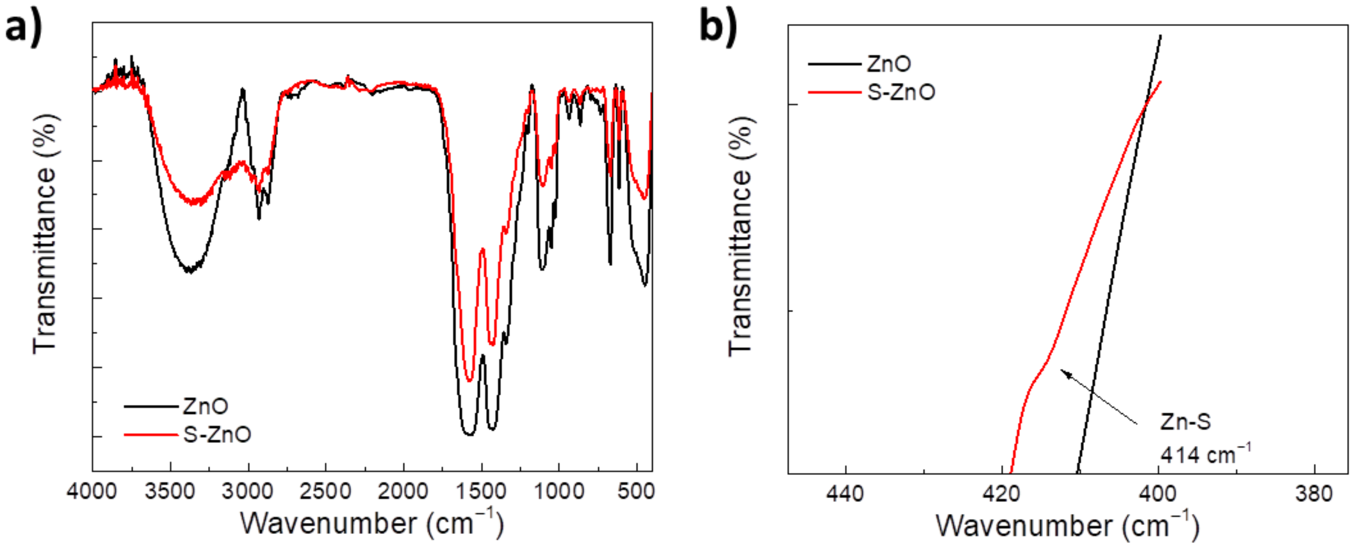

Figure S1: a) FTIR transmittance spectra and b) enlarged area of the same spectra of pristine and sulfurized ZnO annealed at 250°C for 30 min.

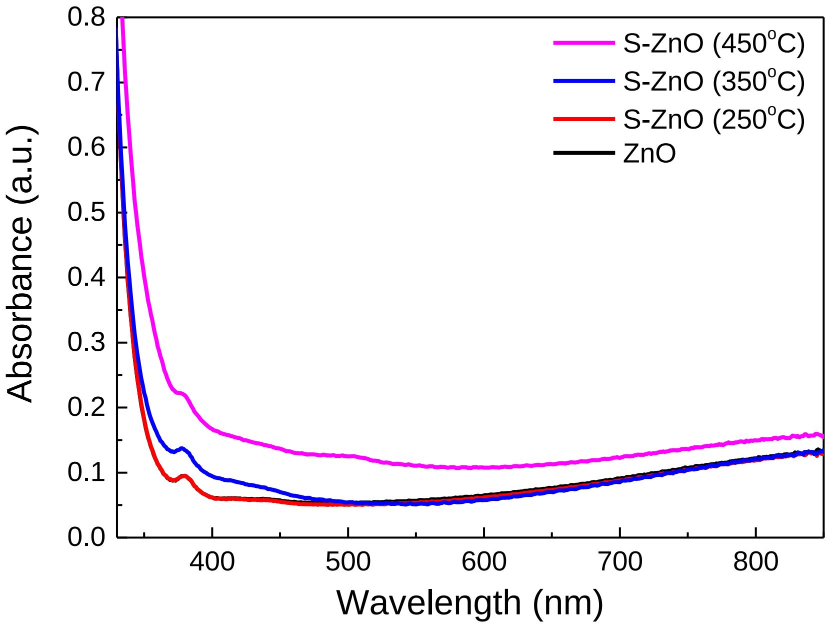

Figure S2: UV-Vis absorption spectra of pristine and sulfurized ZnO thin films annealed at various temperatures for 30 min.

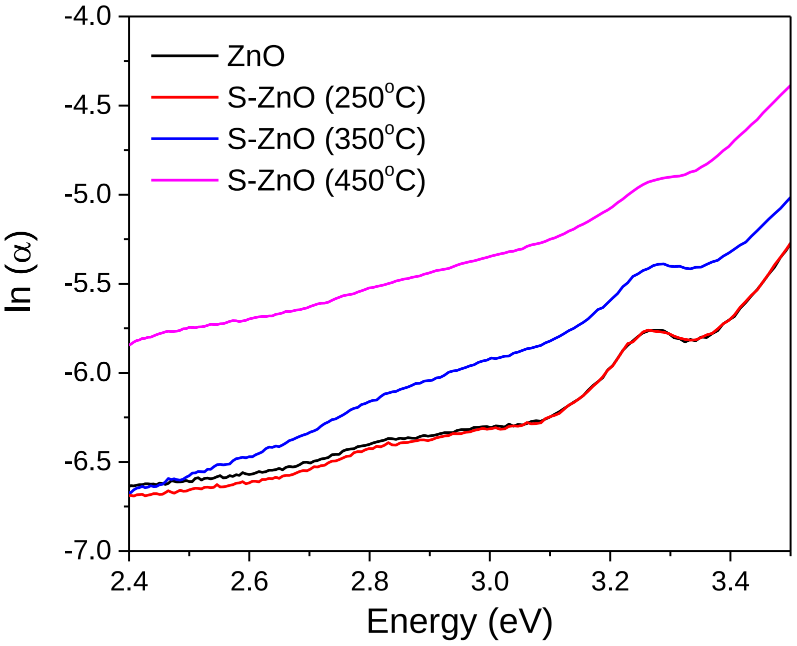

Figure S3: Urbach energy of pristine and sulfurized ZnO thin films annealed at various temperatures for 30 min.

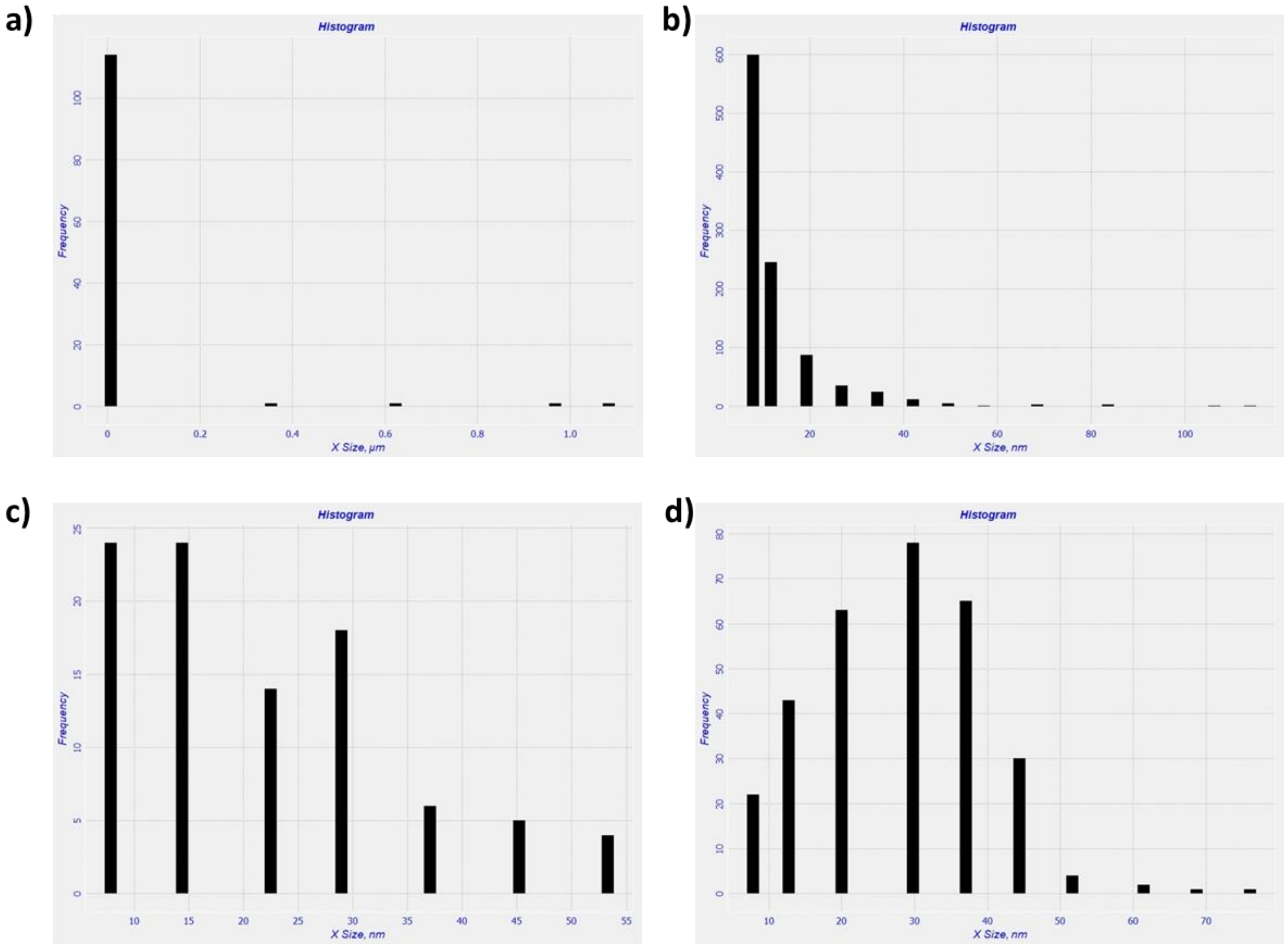

Figure S4: Histogram of grain size of a) ZnO, b) S-ZnO (250ºC), c) S-ZnO (350ºC) and d) S-ZnO (450ºC) substrates.

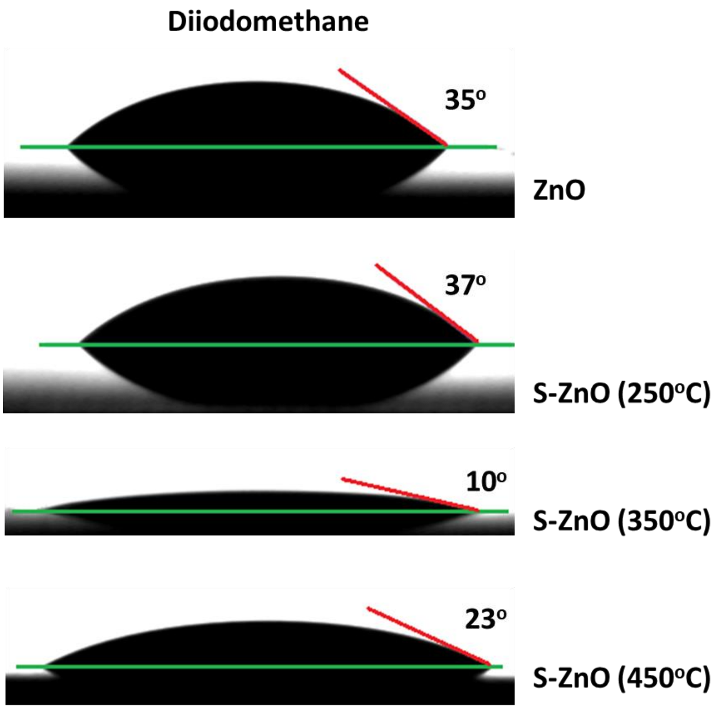

Figure S5: Contact angle measurements of a droplet of diiodomethane on ZnO and S-ZnO substrates annealed at various temperatures for 30 min.

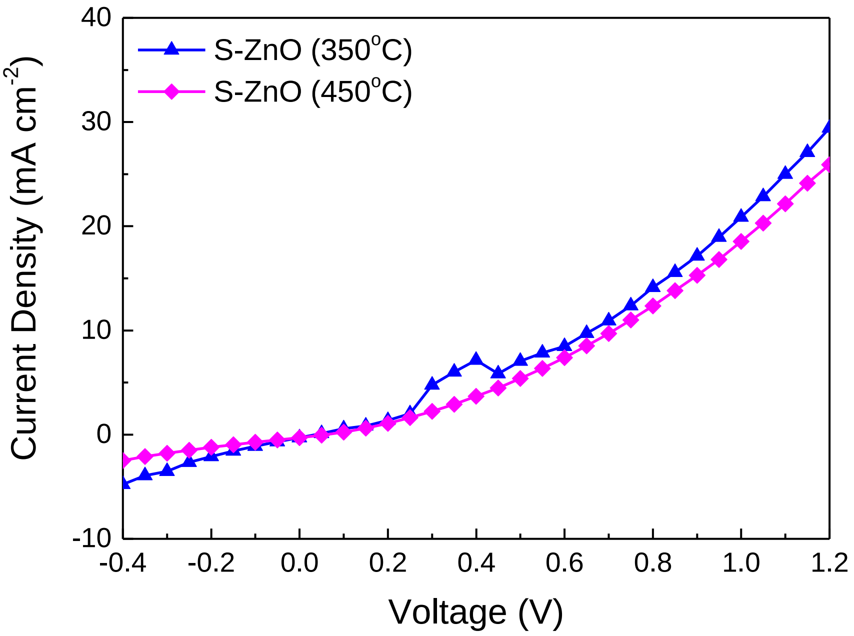

Figure S6: Current density – voltage (J – V) characteristic curves of inverted OSCs based on S-ZnO EELs annealed at 350°C and 450°C for 30 min.

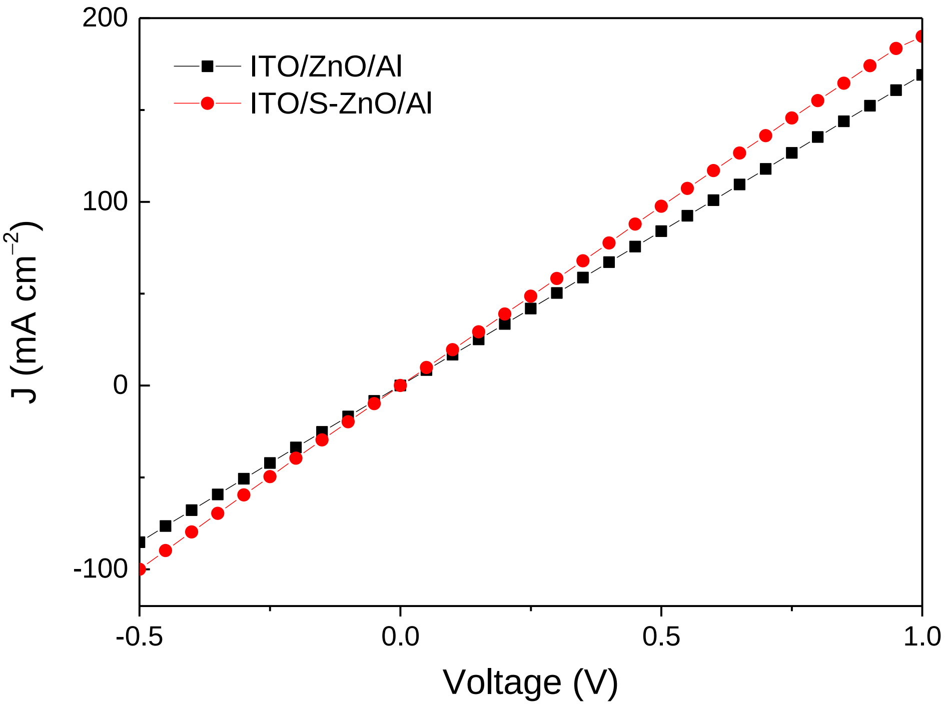

Figure S7: J−V curves of the devices with the architecture ITO/ZnO or S-ZnO/Al.

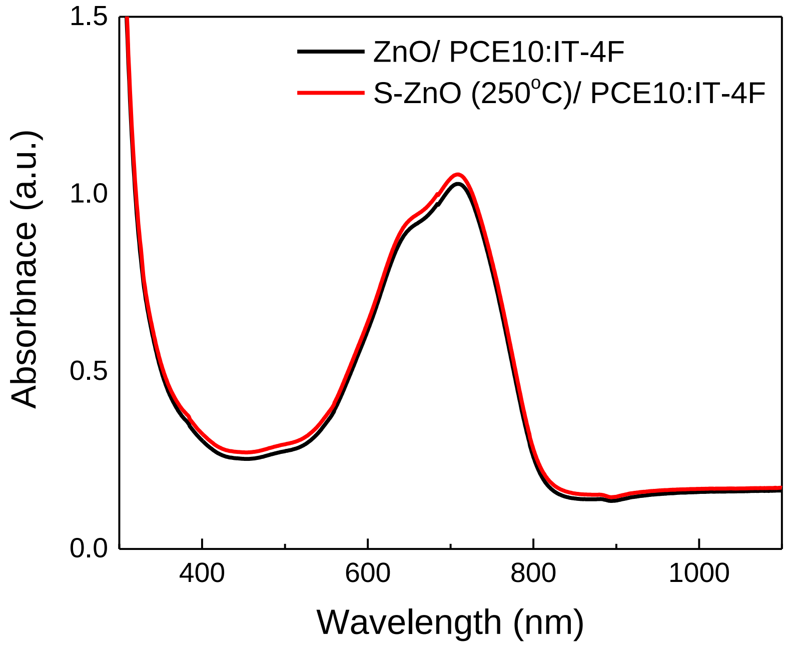

Figure S8: UV-Vis absorption spectra of PCE10:IT-4F spin-coated on pristine and sulfurized ZnO thin films annealed at 250°C for 30 min.

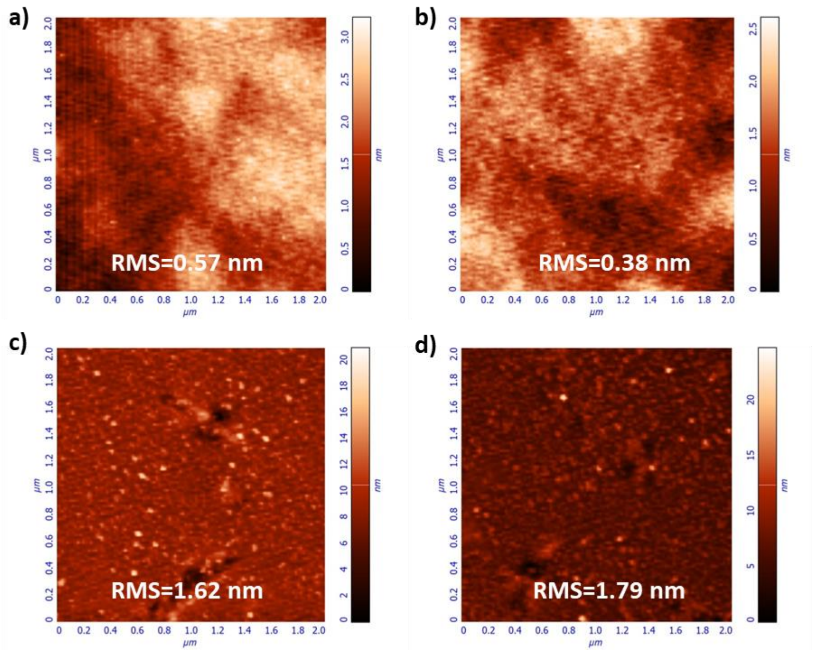

Figure S9: 2x2 μm^2 AFM height images of PCE10:IT-4F spin-coated on a) ZnO, b) S-ZnO (250°C), c) S-ZnO (350°C), and d) S-ZnO (450°C) substrates.

Table S1: XRD analysis showing d-spacing and crystalline size (D) of ZnO and S-ZnO annealed at 250°C for 30min.

**Sample**	**(hkl)**	**2θ (deg)**	**FWHM (rad)**	**d (nm)**	**D (nm)**
ZnO	(100)	31.88	0.016	0.280	8.94
	(002)	34.43	0.013	0.260	11.61
	(101)	36.23	0.016	0.247	9.63
S-ZnO (250°C)	(100)	31.76	0.014	0.281	10.11
	(002)	34.46	0.012	0.260	12.19
	(101)	36.05	0.014	0.249	10.61
S-ZnO (350°C)					
S-ZnO (450^{oC})	(100)	31.67	0.015	0.282	9.28
	(002)	34.49	0.014	0.259	9.85
	(101)	35.99	0.014	0.249	10.35

Table S2: XRD analysis showing lattice parameters (α and c), unit cell volume (V), and bond length (L) of ZnO and S-ZnO annealed at 250°C for 30min.

**Sample**	**α_{100} (Å)**	**c_{002} (Å)**	**V (Å)**	**L (Å)**	**δ (·10^{-4} nm^{-2})**
ZnO	3.233	5.203	47.23	1.972	12.5
S-ZnO (250°C)	3.249	5.199	47.54	1.976	9.7
S-ZnO (350°C)	3.246	5.211	47.65	1.978	7.5
S-ZnO (450°C)	3.258	5.194	51.89	1.980	11.6

Table S3: Energy dispersive X-ray spectroscopy (EDX) analysis for pristine and S-doped ZnO samples annealed at various temperatures.

**Sample**	**Element**	**Wt %**	**Atomic %**
ZnO	Z	71.59	38.14
	O	28.41	61.86
S-ZnO (250°C)	Z	73.54	40.65
	O	26.10	58.95
	S	0.36	0.40
S-ZnO (350°C)	Z	79.00	53.51
	O	12.61	34.91
	S	8.38	11.58
S-ZnO (450°C)	Z	76.34	52.19
	O	10.60	29.61
	S	13.06	18.21

Table S4: Calculated values of band gap and Urbach energy of pristine and S-doped ZnO films annealed at various temperatures.

**Sample**	**E_g (eV)**	**E_u (eV)**
ZnO	3.45	0.305
S-ZnO (250°C)	3.45	0.311
S-ZnO (350°C)	3.38	0.424
S-ZnO (450°C)	3.22	0.509

Table S5: Surface energy as derived from contact angle measurements of ZnO and S-ZnO substrates.

Sample	θ_w (º)	θ_d (º)	γ_{sp} (mJ m^{-2})	γ_{sd} (mJ m^{-2})	γ (mJ m^{-2})
ZnO	69.8	35	8.20	37.31	45.51
S-ZnO (250ºC)	52.4	37	19.60	33.41	53.01
S-ZnO (350ºC)	32	10	28.01	39.85	67.86
S-ZnO (450ºC)	53.2	23	16.34	39.48	55.82
